# A cross-functional nanostructured platform based on carbon nanotube-Si hybrid junctions: where photon harvesting meets gas sensing

**DOI:** 10.1038/srep44413

**Published:** 2017-03-15

**Authors:** F. Rigoni, C. Pintossi, G. Drera, S. Pagliara, G. Lanti, P. Castrucci, M. De Crescenzi, L. Sangaletti

**Affiliations:** 1 Surface Science and Spectroscopy Lab @ I-Lamp and Dipartimento di Matematica e Fisica, Università Cattolica del Sacro Cuore, Brescia, Italy; 2 CNR-INO and Sensor Lab, Dept. of Information Engineering, University of Brescia, Italy; 3 Dipartimento di Fisica, Università di Roma, Tor Vergata, Italy

## Abstract

A combination of the functionalities of carbon nanotube (CNT)-Si hybrid heterojunctions is presented as a novel method to steer the efficiency of the photovoltaic (PV) cell based on these junctions, and to increase the selectivity and sensitivity of the chemiresistor gas sensor operated with the p-doped CNT layer. The electrical characteristics of the junctions have been tracked by exposing the devices to oxidizing (NO_2_) and reducing (NH_3_) molecules. It is shown that when used as PV cells, the cell efficiency can be reversibly steered by gas adsorption, providing a tool to selectively dope the p-type layer through molecular adsorption. Tracking of the current-voltage curve upon gas exposure also allowed to use these cells as gas sensors with an enhanced sensitivity as compared to that provided by a readout of the electrical signal from the CNT layer alone. In turn, the chemiresistive response was improved, both in terms of selectivity and sensitivity, by operating the system under illumination, as the photo-induced charges at the junction increase the p-doping of CNTs making them more sensitive to NH_3_ and less to NO_2_.

In the last years experiments on hybrid heterojunctions between carbon nanotubes (CNTs) and Si wafers have demonstrated the possibility to achieve low-cost, high efficiency devices for photovoltaic (PV) applications^1^,^2^,^3^,^4^,^5^,^6^,^7^,^8^,^9^,^10^,^11^,^12^,^13^,^14^,^15^,^16^,^17^,^18^,^19^. The hybrid CNT-Si architectures can be ranked among the most promising systems for the next generation PV with power conversion efficiency (PCE) up to 17%^10^.

In these cells, the p-type Si layer (the so-called emitter) is replaced by a semi-transparent CNT film deposited at room temperature on the n-doped Si wafer, thus giving rise to an overall reduction of the total Si thickness and to the fabrication of a device with inexpensive methods at low temperatures. In particular, the CNT film deposited onto the Si wafer both acts as a conductive electrode and establishes a built-in voltage for separating photocarriers. Moreover, due to the CNT film optical semitransparency, most of the incoming light is absorbed by Si; thus the efficiency of the hybrid CNT-Si solar cell may in principle be comparable to a conventional Si p-n junction solar cell.

CNTs are also well known as the building block of many gas sensing devices^20^,^21^,^22^,^23^,^24^,^25^,^26^,^27^,^28^,^29^,^30^,^31^,^32^,^33^,^34^,^35^,^36^,^37^,^38^,^39^,^40^. Gas interaction with CNTs is known to produce remarkable resistivity changes, depending on the oxidizing or reducing nature of the gas. For instance, the exposure of p-type CNTs to oxidizing NO_2_ molecules reduces the CNT resistivity^41^, while the resistivity is increased when the CNTs are exposed to reducing gases such as NH_3_. In the last decade, CNTs have been indeed widely studied as sensitive materials to produce gas sensors, both in chemiresistor and chemical field-effect transistor (chem-FET)^27^,^28^,^30^,^31^,^38^ configurations. As schematically shown in Fig. 1-a,b, the main difference in the operational features is that in a chemiresistor (Fig. 1-a) the sensor resistance (or the current which flows through the CNTs between source and drain electrodes) and the resistance variation due to the presence of chemical species are monitored, while in a chem-FET configuration (Fig. 1-b), the current flowing through the CNTs can be also steered by a third electrode (the gate electrode), generally located below the CNT film.

The electronic properties and the behaviour of a chem-FET based on CNTs are well known^42^. In particular, Uchida *et al*.^43^ discussed through a first-principles study the variation of the Fermi level upon changing the gate potential. The results indicate that with a positive gate potential extra electrons are injected into the CNT layer and the Fermi level E_F_ shifts upwards; while with a negative gate potential extra holes are injected into the CNT layer and E_F_ shifts downwards (Fig. 2a–c).

So far the interplay between the photon harvesting and gas sensing functionalities of CNTs has virtually been neglected, but the nature of the junctions and the morphology of the CNT layer (usually made of CNT bundles), may disclose new potentialities. In particular the CNT-Si junction prepared by using a layer of CNT bundles can be regarded as an open system: for example chemical interactions are known to affect also the CNT-Si interface and ultimately the cell properties through selective etching or optimal oxidation of the buried SiO_x_ layer at the interface^13^,^44^,^45^. Etching with HNO_3_ or HF is known to selectively tailor the thickness and the chemical properties of the buried SiO_x_ layer, allowing to explore its role in determining the PV cell efficiency. These effects are usually permanent along the PV cell life time. Recently, temporary effects of environment on the PV cell performances were also investigated by exposing the cells to air, O_2_, and water vapour^46^ and N_2_ flow^47^, though changes were mainly related to relative humidity and cooling effects of the gas flowing on the layers. In particular, Fan *et al*.^47^ investigated the photovoltaic behaviour of CNT-Si solar cells under N_2_ gas flow, showing that a change in the cell temperature induced by the gas flow can lead to an enhancement in the cell efficiency, thereby suggesting that these cells can act as gas flow sensors while generating energy through the photovoltaic effect.

Devices that combine energy generation and other specific functions are also highly desirable for the development of wearable applications. Self-powered sensors with both energy-harvesting and sensing capabilities in a single device have recently drawn much attention for application in wearable devices. For instance, energy-harvesting devices can be directly employed for the detection of mechanical and thermal stimuli, leading to self-powered sensors^48^,^49^.

In the case of devices based on CNTs, hybrid CNT-Si cells have been used as pressure sensors^50^, or wind sensors^47^.

Self-powering and wearability are two features that could be matched by CNT-Si devices, provided that the devices can be assembled on flexible substrates. The fabrication of flexible PV cells based on the CNT-Si junction has been recently demonstrated by Sun *et al*.^51^. This result discloses the possibility to use CNT-Si junctions to develop self-powered, wearable, gas sensing devices working at room temperature. In this case, the PV cell functionality can power the gas sensing functionality, which does not require the power-consuming modules typical of metal-oxide based gas sensors that need to be operated at high temperatures through additional heating elements.

In the present study we explore the interplay between photovoltaic and gas sensing functionalities in CNT-Si based PV cells. We demonstrate that (i) cross-functionality can enable further developments of the CNT-Si PV cells, and that (ii) the selectivity and sensitivity of CNT-based gas-sensing devices can be improved by coupling these layers with n-doped Si.

In particular, we demonstrate the possibility to tune the cell efficiency by reversible interaction with selected gas (namely NH_3_ or NO_2_), and the possibility to use the PV devices as chem-FET, driven by light absorption, for the selective detection of polluting gases. Furthermore, as the open circuit voltage (V_OC_), the short circuit current (I_SC_), the fill factor (FF), and the peak power (PP) parameters of the PV cell showed to be quite sensitive to gas exposure, the readout of these parameters alone provides a way to use the PV cell directly as a gas sensor, with sensitivities that in some cases were higher than those registered with the chemiresistive read-out scheme.

## Results

**Figure 1:**
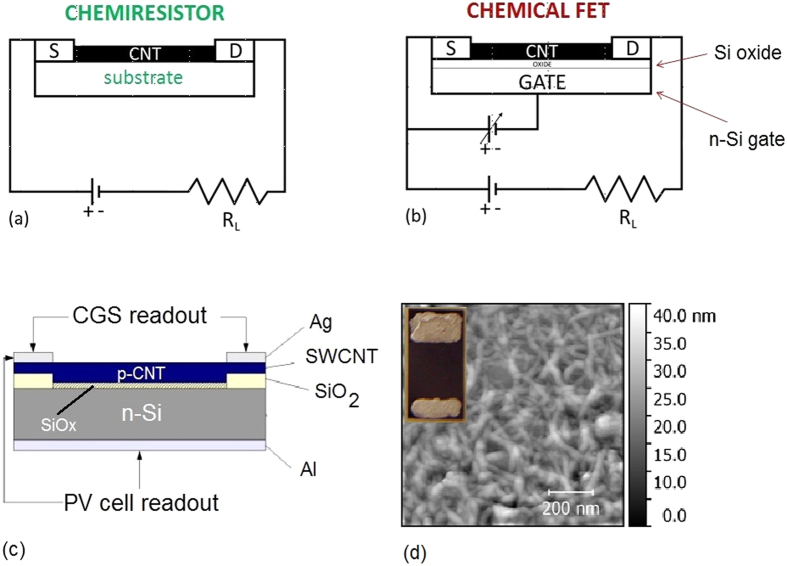
Read-out schemes for electrical measurements in a chemiresistor (**a**) and chem-FET (**b**) configurations. Chemiresistor gas sensor and PV cell read-out schemes of the present SWCNT-Si device (**c**). AFM scan of the Cell 15 surface (1 × 1 μm^2^). Inset: top view of Cell 15 with the two Ag parallel electrodes (**d**).

This study is based on two CNT-Si based PV cells prepared by depositing a single-walled CNT layer in contact with an n-type Si wafer to obtain a hybrid CNT-Si junction. The schematic layout of the hybrid CNT-Si cell is shown in Fig. 1-c, where the different layers of the PV cells are evidenced. In one cell, hereafter denoted as Cell 15, the thickness of the CNT bundle layer is 25.0 ± 1.2 nm, while in the other, hereafter denoted as Cell 55, has a thicker CNT bundle layer of 45.0 ± 1.5 nm. Upon fabrication, these cells displayed a PCE of 0.26% and 1.31%, respectively. These are not record efficiencies, as more efficient cells have been produced by using the same method and improving the overall device assembly and the quality of the CNT layer (see, e.g. ref. 52), but they were chosen because the CNTs were contacted with two parallel electrodes that enable also a chemiresistive readout of the signal along the CNT bundle layer. The main features of the cells are summarized in Table 1. AFM scan of the Cell 15 and the overall view of the PV cell with the two parallel electrodes is shown in Fig. 1-d.

### Reversible Steering of Efficiency

A systematic study of the PV cell characteristics upon exposure to oxidizing (NO_2_), reducing (NH_3_), or weakly interacting gases (acetone, ethyl alcohol) has been carried out through measurements of the I-V curves under different ambient conditions. As expected, the effects of weakly interacting gases on the I-V characteristics were negligible (with PV cell parameters variations below 1% of the values recorded before exposure), therefore in the following we will focus on the effects of NH_3_ and NO_2_. Cell cycling through multiple gas exposure/recovery processes resulted in a good cell stability. An example of cycling is shown in Fig. S1 of the Supplementary Information, where the V_OC_ variation of Cell 55 is tracked as a function of time during the exposure processes.

To highlight the main features of the I-V curves and the possible changes induced by gas adsorption, the I-V data collected from both cells upon exposure up to 2 ppm NO_2_ are shown in Fig. 3, along with the corresponding Power(VI)-V curves.

As can be observed, the cell parameters are clearly affected by the exposure to gas, though at different extents, depending on the cell type. In addition to changes of the overall I-V curve shape, the most evident change can be retrieved in the V_OC_ and I_SC_ values. Changes in V_OC_ and I_SC_ are known to occur in PV cells when an external load is connected to the cell, either in series or in parallel, to the series resistance R_S_ and to the shunt resistance R_Sh_, respectively. This means that gas exposure is mainly acting on the R_S_ or R_Sh_ values of the PV cell, allowing one to steer these resistances and therefore the electrical behaviour of the PV cell.

This assumption is consistent with the behaviour observed when an external load was systematically applied to both cells, either in series or in parallel, and changes in the I-V curve were tracked. The results (shown in the Supplementary Information, Fig. S2) indicate that the overall behaviour resembles that expected for a PV junction, as described in many textbooks^53^. For both cells, the increase of R_S_ determines a drop of I_sc_, while V_OC_ is constant. In turn, the increase of R_Sh_ determines in both cells an increase of V_oc_.

The effects of charge injection or extraction (depending on the adsorbed molecule) on R_S_, R_Sh_, and on the FF can be accounted for by modelling the PV cell with the one-diode scheme shown in Fig. 4 (refs 54 and 55), that is suitably modified to account for the effect of gas exposure with additional, variable, R_S_ and R_Sh_ loads.

The equation (Eq. 1) underlying this scheme is:


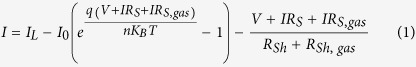


where R_S_ and R_Sh_ are now corrected by the terms R_S,gas_ and R_Sh,gas_ that are dependent on the gas concentration.

The I-V curves upon gas exposure have been tracked spanning a time window of several hours, depending on the recovery time of the initial conditions at room temperature. The time window includes ten minutes before gas exposure, the gas exposure itself, and the recovery time. Each I-V curve typically takes 25 seconds to be automatically measured by our I-V tracker. The gas concentration during each exposure is measured with a calibrated chemiresistor.

In Figs 5 and 6 the behaviour of V_OC_, I_SC_, PP, FF of the PV cells is shown as a function of time across the exposure to NH_3_ (Fig. 5) and NO_2_ (Fig. 6). The time window of gas exposure is marked by shaded areas. The main results are also summarized in Table 2. In this Table the results are reported as a relative variation (ΔI_sc_/I_sc,0_, ΔV_oc_/V_oc,0_, ΔFF/FF_0_, ΔPP/PP_0_) with respect to the parameter values before gas exposure.

In principle, if charge injection or extraction occur during gas adsorption on the CNT bundle layer, the PV cell efficiency is expected to be affected by changes of the CNT charge carrier density. In the case of NH_3_, an increase of R_S_ for both cases is expected, since NH_3_ behaves as an electron donor with respect to p-doped CNTs. This is confirmed by the data shown in Fig. 5. Along with a reduction of I_sc_ (ΔI_sc_/I_sc,0_ = −15% and −1.3% for Cell 15 and Cell 55, respectively) expected from an increase of R_S_, also a V_oc_ reduction is observed for both PV cells (ΔV_oc_/V_oc,0_ = −13% and −4.0% for Cell 15 and Cell 55, respectively). Larger effects on the PV cell parameters are observed for Cell 15. It is also interesting to observe that while for Cell 15 (Fig. 5-left panel) the I_sc_ and V_oc_ values recover at room temperature to those measured before exposure, in the case of Cell 55 (Fig. 5-right panel) the exposure to NH_3_ induces an increase of PP, that does not show a recovery after 200 minutes, in spite of the recovery of both I_sc_ and V_oc_.

Exposure to NO_2_ results in a quite different behaviour (Fig. 6). For Cell 55 (Fig. 6-right panel), interaction with NO_2_ yields an increase of I_sc_ (ΔI_sc_/I_sc,0_ = +0.1%), as expected from the decrease of R_S_ related to p-doping, but a decrease of V_oc_ (ΔV_oc_/V_oc,0_ = −1.2%). The FF decreases (ΔFF/FF_0_ = −2%), along with the PP (ΔPP/PP_0_ = −0.5%) and, ultimately, the PCE, defined as the ratio between PP and the incident power P_inc_ (60 mW/cm^2^ provided by an halogen lamp). However, the cell does not recover quickly to the initial conditions, indicating that interaction with NO_2_ does not produce reversible effects on a short timescale. In the case of exposure to NO_2_ the recovery can be assisted by UV illumination, as discussed in ref. 56.

In the case of Cell 15, the effects of p-doping on the CNT layer are confirmed by the I_sc_ increase (ΔI_sc_/I_sc,0_ = +17%). Also V_oc_ increases (ΔV_oc_/V_oc,0_ = +6.4%), along with PP (ΔPP/PP_0_ = +28%), resulting in a remarkable increase of PCE.

Therefore, we show that the PCE can be steered by interaction with molecules and that changes in PCE can be reversible on different time scales, depending on the adsorbed gas and on the thickness of the CNT bundle layer. Namely, the initial conditions can be restored more easily in the device with the thinner layer (Cell 15) and after exposure to NH_3_. Exposure to NO_2_ yields a PCE increase in Cell 15 but with a longer recovery time at room temperature with respect to the NH_3_ case.

It is important to observe that changes in PV cell parameters upon gas exposure are themselves a way to use these PV cells as gas sensors. Therefore a comparison with the results obtained in the chemiresistive read-out scheme (Fig. 1-a) is also reported in Table 2. These results are expressed in terms of the (ΔR/R)/[NH_3_] and (ΔR/R)/[NO_2_] sensitivity values, where [NH_3_] and [NO_2_] are the NH_3_ and NO_2_ concentrations during the exposure, respectively. A remarkable increase of sensitivity ΔI_sc_/I_sc_/[gas] with respect to the chemiresistor readout configuration is registered in Cell 15 after exposure to both NH_3_ (from 0.153 to 0.500, absolute value) and NO_2_ (from 6.74 to 8.50, absolute value), whereas the best performance for Cell 55 is registered in the chemiresistor configuration. This suggests that the thinner CNT layer of Cell 15 (25 nm) could favour the effects that determine the variation of electrical parameters across the junction, while in Cell 55 these effects are weakened by the relatively thicker CNT layer (45 nm). Indeed, the dependence of the cell PCE on the CNT layer thickness shows a well-defined behaviour^13^,^57^,^58^,^59^. PCE increases with thickness up to an optimal value (e.g. 32 nm in cells similar to the present ones^59^) and then drops for larger thickness values. The maximum value of the PCE is interpreted in terms of competition between the nanotube film optical transparency, the total number of nanotube/Si heterojunctions (i.e. the Si surface area covered by CNT) and nanotube network resistance^13^. The present cells are, respectively, too thin (Cell 15) and too thick (Cell 55) to present enhanced response to gas directly related to an enhanced PCE. Therefore, we ascribe the gas effect on the PV cell parameters to the distance of the CNT surface layer from the interface, i.e. the smaller the distance, the larger the effects.

It is also important to note that while ΔI_sc_/I_sc,0_ changes remarkably in Cell 15 after exposure to NH_3_ and NO_2_ (−15%, +17%, respectively), even larger changes are detected for the ΔPP/PP_0_ parameter (−23%, +28%, respectively). This implies that the gain observed with respect to the chemiresistive configuration can be further enhanced if ΔPP/PP_0_ is tracked upon gas exposure rather than ΔI_sc_/I_sc_.

### Optically driven-ChemFET

**Figure 2:**
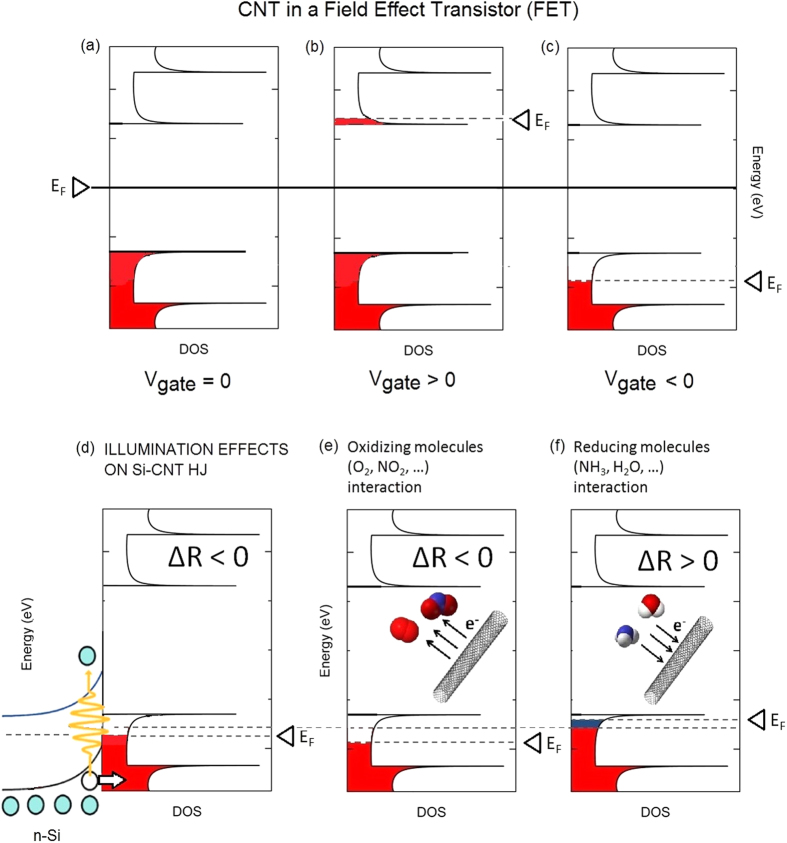
Top panel: Schematic of V_gate_ induced variation of E_f_ in a CNT-based FET (**a**–**c**). Bottom panel: (**d**) Qualitative scheme of light effect on the CNT density of states (DOS) in a Si-CNT heterojunction (HJ). Upon photon absorption in n-Si, holes are injected into the VB of CNT, increasing the number of carries and thereby decreasing the resistance (ΔR < 0) in the p-doped CNT layer. (**e**,**f**) Schematic representation of the effects of gas interactions with CNTs at the basis of gas sensing in p-doped CNT-based chemiresistors: (**e**) interaction with oxidizing molecules increases the p doping and decreases the resistivity of p-type CNT (ΔR < 0); (**f**) interaction with reducing molecules decreases the p doping and increases the resistivity (ΔR > 0).

So far the measurements (I-V tracking curves or chemiresistor read-out) have been carried out under ambient light. In order to investigate the extent of photo-effects on the gas sensing properties we measured the current through the chemiresistors both in dark conditions and under illumination. At this stage we added a chemiresistor made with a CNT bundle layer deposited onto an alumina substrate with interdigitated electrodes. This device is just a conventional, non-FET, CNT-based chemiresistor, to be used as benchmarking to discuss the origin of photoinduced effects. Upon illumination, in the PV cells, holes are transferred from Si to CNTs [see, e.g., ref. 7 and Refs. therein] and therefore, as the gate voltage in a FET, the intensity of the light source (triggering the photoabsorption in Si) is expected to control and shift the Fermi level in the CNT density of states (Fig. 2d). Indeed, the overall cell structure can be described as a FET, where the gate potential is represented by the built-in potential of the p-n heterojunction. It is important to recall that CNT-Si cells based on a p-type CNT bundle layer can be regarded as MIS^9^,^44^, as they can display a Si oxide layer at the interface between the CNT bundle layer and the n-Si. This justifies the choice to describe these junctions as a FET architecture.

In a chemiresistor configuration, charges flow in the CNT layer, where two electrodes on top of the CNT layer collect the in-plane current, but their flow can be affected by the potential at the junction. If not driven by the n-type Si back-contact, in principle this potential is fixed, depending on the characteristics of the junction. However, charge carriers can be created at the junction through illumination at different wavelengths and intensities. This allows to drive the gate-equivalent potential through optical absorption. As CNT-based FET are known to be able to detect gas molecules adsorbed on the CNT surface (acting therefore as chem-FETs^30^), the present devices can be operated as chem-FETs driven by photon absorption.

**Figure 3:**
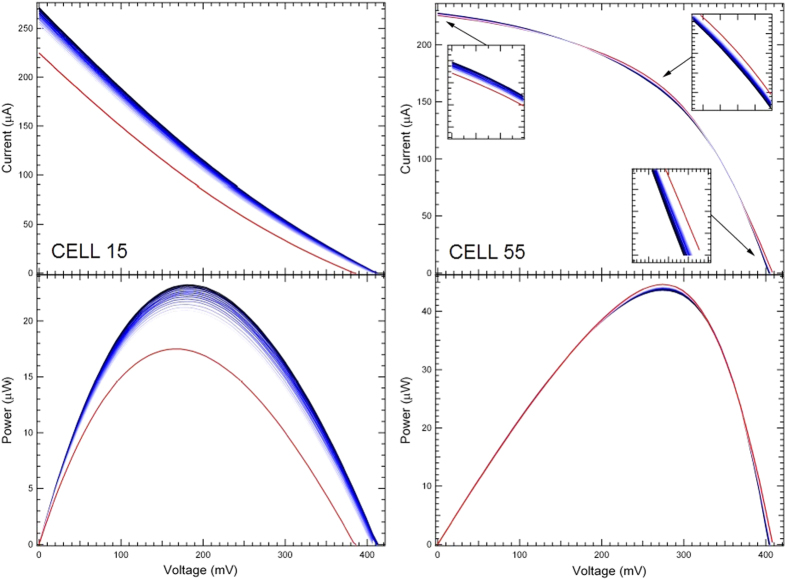
Tracking of I-V curves of Cell 15 (left panel) and Cell 55 (right panel) upon exposure to up to 2 ppm NO_2_ (blue lines) For both cells a data set of I-V curves and the corresponding IV-V curves are displayed. Red curves correspond to 0 ppm NO_2_.

**Figure 4:**
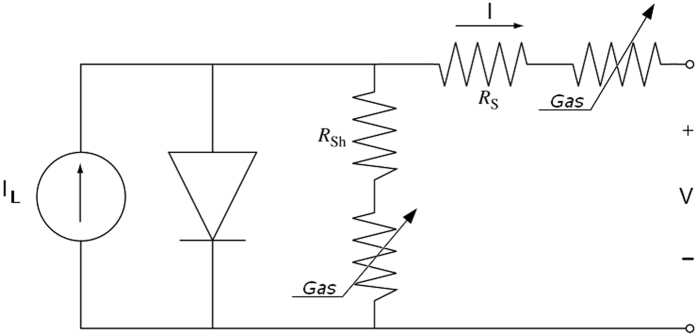
Scheme of the PV cell, suitably modified to account for the effect of gas exposure with additional, variable, R_s_ and R_Sh_ loads.

**Figure 5:**
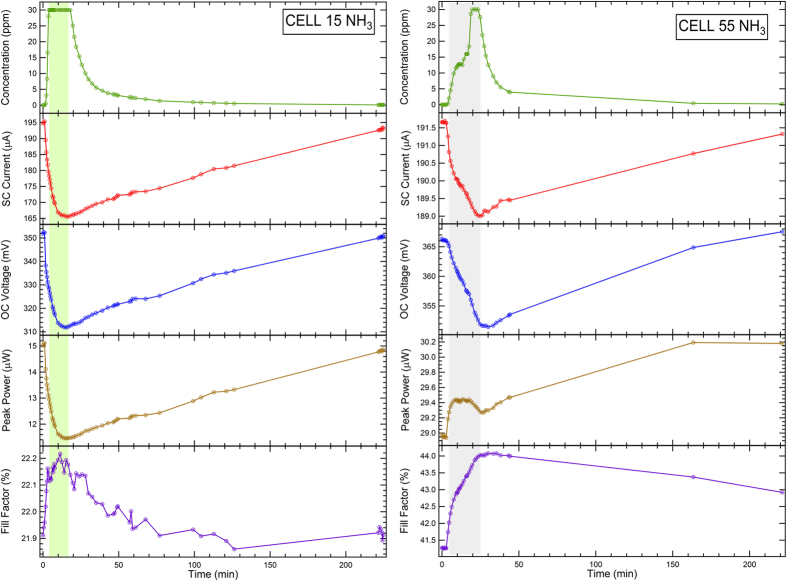
Effects on Cell 15 (left) and Cell 55 (right) of the exposure to NH_3_. The shaded areas represent the exposure time. The top panels show the NH_3_ concentration. The maximum NH_3_ concentration was 30 ppm.

**Figure 6:**
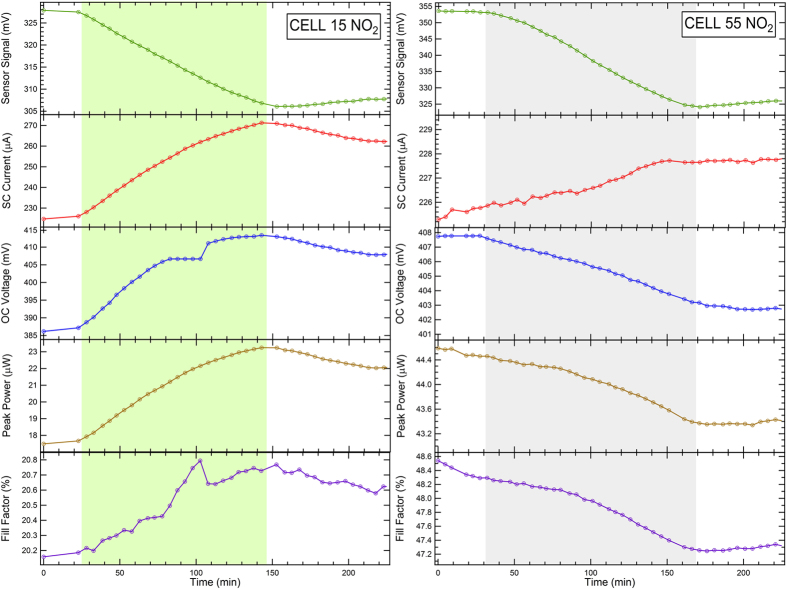
Effects on Cell 15 (left) and cell 55 (right) of the exposure to NO_2_. The shaded areas represent the exposure time. The top panels show the reference sensor signal. The maximum NO_2_ concentration was 2 ppm. The jump in V_OC_ and FF registered at time = 100 s for Cell 15 is an experimental artefact.

We tested as first the Cell 55 and Cell 15 devices as gas sensors, under dark conditions, in a chemiresistor configuration, i.e. by detecting the signal value between source and drain electrode and monitoring the resistance (or current) variation during the exposure to NH_3_ and NO_2_ polluting gases. We observed an increase of the sensor resistance in the presence of NH_3_, and a decrease in the presence of NO_2_, as expected for a p-type semiconductor^60^, indicating that the interaction is between gas molecules and the CNT top layer (Fig. 3, left panel). Changes in Cell 15 and Cell 55 response to gas exposure were observed when the same measurements were carried out under illumination, while the reference sensor did not display any appreciable change with respect to the dark conditions. This excludes that the observed changes in the electrical properties of Cell 15 and Cell 55 are due to photocurrents created by photon absorption in the CNT bundle layer.

**Figure 7:**
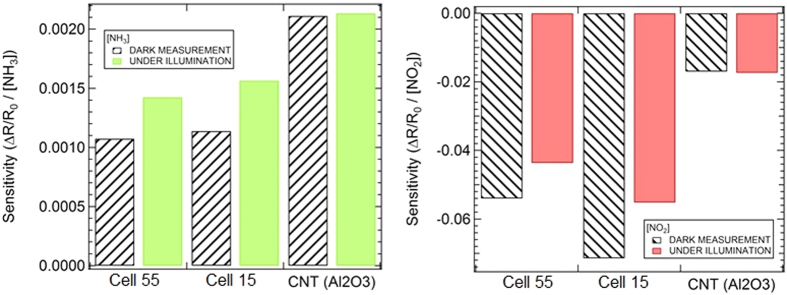
Effects of light on the sensitivity of Cell 15 and Cell 55 operated as chemiresistor to detect NH_3_ (left panel) and NO_2_ (right panel) target molecules.

Results on gas sensing in chemiresistive and chem-FET configurations are summarized in Fig. 7 and Table 3. These data show that, when gas exposure is carried out under illumination conditions, the CNT bundles of both samples react displaying an enhanced sensitivity (+33% and +37% for Cell 55 and Cell 15, respectively) to reducing gases (e.g. NH_3_) and a reduced sensitivity (−19% and −23% for Cell 55 and Cell 15, respectively) to oxidizing gases (e.g. NO_2_). In addition to the sensitivity enhancement, this result also demonstrates a selectivity enhancement, as the effects of the reducing and oxidizing molecules yield opposite changes.

The cell behaviour can be rationalized as follows. Upon illumination holes are injected from the n-side to the p-side of the junction (see Fig. 2-d and ref. 7). This favours the interaction with reducing molecules (e.g. NH_3_) which can more easily transfer electrons to the CNT layer. In turn, the interaction with oxidizing molecules is inhibited, as they find a lower density of states available for charge transfer to the molecules themselves.

## Discussion

### Beneficial effects on PV cells

The exposure to gas during the operation as a PV device clearly shows how the PV cell parameters are strongly affected by the interaction of the device with both reducing and oxidizing molecules. It is likely that these molecules can interact with the device both at the level of CNT bundles and at the CNT-Si interface. In general this interaction is made possible by the porous structure of the CNT layer (see e.g. AFM mapping of Cell 15, Fig. 1-d).

The most important factors that can be considered to discuss the change of PV parameters are the CNT-doping and changes in the CNT work function. As for the first factor, this is clearly visible in Cell 15 where the thin CNT bundle layer interacts with the gas molecules and the extent of CNT p-doping can be modified, depending on the type of molecules: reducing NH_3_ injects electrons and reduce the p-doping, oxidizing NO_2_ extract electron from CNT an increase p-doping. Indeed when the layer is exposed to NH_3_ I_sc_ decreases in both cells, more in Cell 15 than in Cell 55. In turn, exposure to NO_2_ determines an increase of I_sc_ in Cell 15. Effects on Cell 55 are usually weaker than on Cell 15, as the gas may diffuse less through the thicker bundle layer of Cell 55 with respect to Cell 15.

Changes in V_OC_ can be ascribed to changes in the CNT work function. As the present PV devices are usually described in a MIS scheme^9^,^44^, V_OC_ (Equation 2) can be expressed^61^,^62^ as:


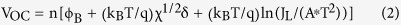


where n is the diode ideality factor, ϕ_B_ is the barrier height, T is the temperature, k_B_ is the Boltzmann constant, δ is the thickness of the insulating layer in the MIS junction, χ is the Si bulk work function, J_L_ is the diode saturation current density, and A* is the Richardson constant.

After CNT p-doping with NO_2_ molecules, an up-shift of the CNT work function occurs and the consequent increase of the barrier height ϕ_B_ yields an increase of V_OC_.

### Beneficial effects on gas sensors

Here a single device can be operated (i) as a conventional chemiresistor, (ii) as a chemiresistor affected by the junction underlying the sensing layer (optical chem-FET), and (iii) as a heterojunction where the I-V curves are affected by gas absorption, thereby providing an electrical output (in terms of V_OC_, I_SC_, FF, and PP) suitable for gas monitoring. The possibility to work in a FET-like condition is remarkable. This configuration is well known for single-CNT FET^63^, one of the most important architectures so far created to probe the transport properties at the nanoscale, but has not been systematically applied to a rougher device where the single CNT is substituted by a CNT bundle layer. The latter set up has the advantage to be much more easily fabricated and has looser requirements on the morphological and electrical features, thereby enabling an easier testing of new architectures and the scale-up to large devices.

In gas sensing functionality we registered the following improvements:

(i) Higher sensitivity to NH_3_ and NO_2_ when the device is operated with the PV cell readout scheme (Cell 15); (ii) Higher sensitivity to NH_3_ (Cell 15, Cell 55) when the devices are operated under illumination; (iii) Higher selectivity to NH_3_ vs. NO_2_ as interfering gas by operating both cells under illumination and in dark conditions.

On the basis of the results so far presented, this study establishes a starting point to explore different kinds of CNT-Si based PV cells, where (i) different classes of CNTs can be selected (e.g., double walled CNTs^64^ or multi-walled CNT^52^) and (ii) the device architecture can be improved to obtain a higher PCE. Our preliminary study on a similar cell (semiconducting CNT, thickness 28 nm, PCE = 6.25%) where the significant increase in PCE was mainly achieved by replacing the parallel electrodes contacts with a single frame electrode shows that gas effects (namely NO_2_ exposure) on the cell behavior are still observed, and an increase of the PCE of about 50.4% is registered. Unfortunately these contacting scheme cannot be used to test the cell as a chemiresistor. However if we consider, in addition to the PCE increase, also the relative variations ΔV_OC_/V_OC_ (+14.5%) and ΔI_SC_/I_SC_ (+2.4%) upon gas exposure, the results indicate that also this PV cell can be used as a gas detector in the PV-cell readout scheme (Fig. 1-b) by measuring the relative change of, e.g., V_OC_ and PCE that results to be the parameters mostly affected by the interaction with target gas molecules.

### Concluding remarks

CNT-Si heterojunctions were considered as an «open» system to build PV devices that can be tailored and improved through interaction with gas molecules at the interface. A reversible steering of PV cell parameters has been demonstrated through absorption of reducing or oxidizing gas molecules on the CNT bundle layer. For the PV device with thinner CNT layer (Cell 15), an increase of efficiency (tracked through an increase of ΔPP/PP_0_ = 28%) has been recorded upon exposure to NO_2_. This is ascribed to an effective p-doping of the cell induced by the adsorption of the oxidizing molecule. Moreover, CNT-Si heterojunctions can be regarded as a playground to explore novel concepts in PV/gas sensing.

A general benchmarking with other systems is generally hindered by the different device architecture (contacting pads, layer thickness) and materials selection (CNT layer preparation, Si doping, thickness of Si wafer, thickness of the SiO_x_ layer). However, in gas sensing applications the possibility to use different read-out schemes allows one to select the scheme where major changes upon gas absorption occurs, exploiting at their best the cross-functional properties of the device.

In particular, the possibility to choose the read-out configuration which yields the best performances for gas sensing is demonstrated, both in terms of sensitivity and selectivity. For the thinner layer (Cell 15) the response read-out through the cell parameters yielded an increased sensitivity to both NO_2_ and NH_3_ as referred to the variation of ΔI_sc_/I_sc,0_, and even larger if referred to ΔPP/PP_0_. The FET-like operation under illumination in a chemiresistive readout configuration has shown that both sensitivity and selectivity can be tuned: under illumination both devices are more sensitive to NH_3_, while readout in dark conditions enhances the sensitivity to NO_2_.

Further implications of this study can be envisaged by considering gas adsorption on the CNT-layer as a tool to reversibly time-control the doping of the p-type CNT layer, providing further degrees of freedom in the design, engineering and optimization of the CNT-Si hybrid PV cells.

## Methods

### Cell fabrication

In the present study two PV cells have been considered. Both of them have been prepared by using a p-type single-walled CNT (SWCNT) layer in contact with an n-type Si wafer to obtain a hybrid CNT-Si junction. The substrates consisted of 5 × 10 mm^2^ slices of a SiO_2_ passivated (thickness 300 nm) n-type Si(100), with an aluminum ohmic back contact. The oxide layer was patterned by a lithographic process with a positive resist followed by a chemical etching in order to obtain a 5 × 5 mm^2^ bare Si window delimited aside by two SiO_2_ steps. Therefore, the device active area is 5 × 5 mm^2^. The chemical etching was carried out by wetting for 5 min the bare SiO_2_ by a HF/NH_4_F buffer solution.

Networked SWCNT films were obtained from SWCNT powders (diameter d = 0.7−1.4 nm, carbon > 90%, purity ≥ 77%, density 0.09 g/cm^3^) provided by Sigma-Aldrich. For the deposition of the SWCNT film onto the Si patterned substrates, a SWCNT dispersion was prepared by ultrasound treatment of 100 μg of SWCNT powder in a 3 wt% aqueous solution of sodium dodecylsulfate (SDS) (98.5% Sigma Aldrich). After the ultrasound treatment, the dispersion was left to settle down, and the clear supernatant containing unbundled nanotubes was divided from the precipitate (mostly bundled nanotubes) and used to fabricate the film. Once the SWCNT film was cast on the membrane filter, the residual SDS was removed by washing with deionized water and subsequently by an ethanol/methanol/water mixture (15:15:70 in volume). Films with different thickness were obtained by filtering different aliquots in volume of the same solution. The SWCNT film was transferred on the patterned Si substrate flipping it over and pressing the SWCNT coated membrane onto the Si/SiO_2_ surface. The residual cellulose acetate membrane lying on top of SWCNT film was removed by dipping the entire sample in warm acetone and finally rinsing in isopropanol. Finally, two parallel silver painted metallic contacts are created on the SWCNT film just on top of the SiO_2_ steps (inset of Fig. 1-d). The SiO_2_ steps between the metal electrodes and the Si underneath avoid short-circuits causing electron leakage.

The thickness of the CNT layer and the optical transmittance T (%) measured at 550 nm for the two samples are reported in Table 1.

The thickness was estimated according to the method presented in refs 13 and 52, based on a combination of angle-resolved photoemission spectroscopy and optical spectroscopy measurements.

Further details on the cell assembly are reported in ref. 13.

The schematic layout of the hybrid Si-CNT cell is shown in Fig. 1-c, where the different layers of the PV cells are evidenced. The cells have been selected with different thickness of the CNT layer. One cell, hereafter denoted as Cell 15, has a thickness of the CNT bundle layer of 25.0 ± 1.2 nm, while the other, hereafter denoted as Cell 55, has a thicker CNT bundle layer of 45.0 ± 1.5 nm.

After the cell fabrication, the PCE (η_0_) was measured with a solar simulator, in air, under AM 1.5G conditions and the cells displayed a PCE of 0.26% (Cell 15) and 1.31% (Cell 55). In turn, the use of polluting gases (especially NO_2_ which is already irritating for throat and lungs at few ppm concentrations) require to follow a safety protocol. Therefore the measurements during gas exposure were carried out in a gas-tight chamber. Since it was not possible to couple the solar simulator with this chamber, the cell was illuminated through a glass viewport with a halogen lamp resulting in a power on the cell surface of 60 mW/cm^2^, a condition not directly comparable to the AM 1.5 G. Consistently, in Table 1 we have reported the relative PCE variations Δη/η_i_ = (η_NO2_ − η_i_)/η_i_, where η_i_ is the PCE just before exposure to gas and η_NO2_ is the PCE during gas exposure, rather than the PCE absolute values.

The devices were fabricated and preliminarily tested at the Physics Department of the University of Rome - Tor Vergata.

### Response of the CNT layer as an ordinary chemiresistor

In order to check the CNT response to the gas adsorbed on the surface, we carried out measurements of the resistivity change across the CNT layer. As shown in Fig. 2, depending on the target gas, the chemiresistor can display a resistivity increase (when exposed to a reducing gas, such as NH_3_) or a resistivity decrease (when exposed to an oxidizing gas, such as NO_2_).

The variation R_S_/R_0_ of sample resistance R_S_ upon gas exposure with respect to the baseline resistance value R_0_ is measured according to the electrical scheme shown in Fig. 1-c. All sensors and PV devices, including humidity and temperature sensors, will be mounted on a specifically designed circuit board connected to a personal computer through a National Instrument PCIe-6251 data acquisition board. The gas mixing is equipped with three mass flow controllers (MFCs). Two low-flow MFCs for controlling the flow rate of the target gas (10.00 ± 0.78 ppm NO_2_ diluted or 47.1 ± 1.2 ppm NH_3_ both diluted in dry air) and a high-flow MFC for further dilution in dry air. The gas concentration has also been measured with a calibrated, commercially available, chemiresistor gas sensor (Figaro, Mod. TGS 2602). The reliability of this sensor calibration curve is routinely cross-checked by measures in the testing chamber with the calibrated MFCs. Further details can be retrieved in ref. 37.

During the exposure to target gas molecules, the testing chamber was filled by keeping the same flux for both gases. This was done to avoid differences in the response due to a different flux, as gas flow was recognized to affect the cell behaviour^47^. After setting the same gas flow for both target gas molecules, we set the target molecule concentration (30 ppm for NH_3_ and 2 ppm for NO_2_) and continued to fill the chamber until these values were reached. Depending on the characteristics of mass-flow-controllers feeding the chamber, this resulted in a longer filling time for NO_2_ with respect to NH_3_. Gas sensing measurements were carried out at I-LAMP labs of the Università Cattolica del Sacro Cuore, Brescia.

### Response of the overall cell to gas exposure

Current-voltage (I-V) curves were collected during exposure to gas, either in dark or under light illumination. From each I-V curve the short circuit current (I_sc_), the open circuit voltage (V_oc_), the fill factor (FF), and the peak power (PP) were collected and related to the effects of gas exposure. The voltage drop upon gas exposure was measured between the Ag pads on the CNT layer (source) and the Al (Cr-Au) back-electrode below the Si substrate (drain). The I-V curves were collected with a suitably developed I-V tracker based on Labview-driven National Instruments board. Sample illumination was achieved by using a halogen lamp with selectable output power. The different read-out schemes adopted for the FET-like configuration and for the chemiresistor are shown in Fig. 1-c. Current-voltage measurements under different ambient conditions were carried out at the I-LAMP labs of the Università Cattolica del Sacro Cuore, Brescia.

## Additional Information

**How to cite this article:** Rigoni, F. *et al*. A cross-functional nanostructured platform based on carbon nanotube-Si hybrid junctions: where photon harvesting meets gas sensing. *Sci. Rep.* **7**, 44413; doi: 10.1038/srep44413 (2017).

**Publisher's note:** Springer Nature remains neutral with regard to jurisdictional claims in published maps and institutional affiliations.

## Supplementary Material

Supplementary Information

## Figures and Tables

**Table 1:** CNT-layer thickness (nm), PCE (η_0_) defined as PP/P_inc_ and measured after the cell preparation, PCE relative change (η_NO2_ − η_i_)/η_i_ during NO_2_ exposure, optical trasmittivity at 550 nm (%).

Cell	CNT layer Thickness (nm)	PCE (η_0_) (%)	(η_NO2_ − η_i_)/η_i_ (%)	Optical Trasmittivity (%)
Cell 15	25.0 ± 1.2	0.26	+28.0	64
Cell 55	45.0 ± 1.5	1.31	−0.5	30

η_i_ is the PCE value measured before the exposure, η_NO2_ is the PCE value measured during the exposure to NO_2_.

**Table 2:** Variation of PV cell parameters and chemiresistor resistivity during exposure to both NH_3_ and NO_2_.

Cell	Target gas	ΔI_sc_/I_sc,0_ (%)	ΔV_oc_/V_oc,0_ (%)	ΔFF/FF_0_ (%)	ΔPP/PP_0_ (%)	ΔI_sc_/I_sc_/[gas] ppm^−1^	ΔR/R/[gas] ppm^−1^
Cell 55	NH_3_	−1.3	−4	−6.5	−1.0	−0.04	0.133
Cell 15	NH_3_	−15	−13	+1.3	−23	− 0.50	0.153
Cell 55	NO_2_	+0.1	−1.2	−2.0	−0.5	1.10	−4.734
Cell 15	NO_2_	+17	+6.4	+2	+28	8.50	−6.740

All variations in the first 4 columns have been evaluated with respect to the readout just prior exposure. Gas concentration ([gas]) was 30 ppm for NH_3_ and 2 ppm for NO_2_.

**Table 3:** Results, normalized to the gas concentration, obtained for ΔR/R_0_ after exposure to NH_3_ and NO_2_.

Sample	ΔR/R_0_ DARK	NH_3_ exposure	Variation%	ΔR/R_0_ DARK	NO_2_ exposure	Variation%
		ΔR/R_0_ UNDER ILLUM.			ΔR/R_0_ UNDER ILLUM.	
Cell 5.5	0.0011	0.0014	33%	−0.052	−0.044	−19%
Cell 1.5	0.0011	0.0016	37%	−0.069	−0.055	−23%
CNT (Al_2_O_3_)	0.0021	0.0021	—	−0.017	−0.017	—

## References

[b1] Li X., Lv Z. & Zhu H. Carbon/Silicon Heterojunction Solar Cells: State of the Art and Prospects, Advanced Materials 27, 6549–6574 (2015). 26422457 10.1002/adma.201502999

[b2] Harris J. M., Semler M. R., May S., Fagan J. A. & Hobbie E. K. Nature of Record Efficiency Fluid-Processed Nanotube–Silicon Heterojunctions, J. Phys. Chem. C 119, 10295–10303 (2015).

[b3] Tune D. D., Flavel B. S., Krupke R. & Shapter J. G. Carbon Nanotube-Silicon Solar Cells. Adv. Energy Mater. 2, 1043–1055 (2012).

[b4] Castrucci P. Carbon Nanotube/Silicon Hybrid Heterojunctions for Photovoltaic Devices. Advances in Nano Research 2, 23–56 (2014).

[b5] Tune D. D. . The Role of Nanotubes in Carbon Nanotube–Silicon Solar Cells. Advanced Energy Materials 3, 1091–1097 (2013).

[b6] Mc Euen P. L. & Park J. Y. Electron Transport in Single-Walled Carbon Nanotubes. MRS Bull. 29, 272–275 (2004).

[b7] Ponzoni S. . Selective Optical Switching of Interface-Coupled Relaxation Dynamics in Carbon Nanotube–Si Heterojunctions. J. Phys. Chem. C 118, 24110–24116 (2014).

[b8] Shi E. . TiO_2_-coated Carbon Nanotube-Silicon Solar Cells with Efficiency of 15%. Scientific Report 2, 884–889 (2012). 10.1038/srep00884 PMC3504926 23181192

[b9] Jia Y. . Strong and Reversible Modulation of Carbon Nanotube-Silicon Heterojunctions Solar Cells by an Interfacial Oxide Layer. Phys. Chem. Chem. Phys. 14, 8391–8396 (2012). 22573091 10.1039/c2cp23639g

[b10] Wang F. . Considerably improved photovoltaic performance of carbon nanotube-based solar cells using metal oxide layers. Nature Communications 6, 6305 (2015). 10.1038/ncomms7305 25692264

[b11] Jung Y., Li X., Rajan N. K., Taylor A. D. & Reed M. A. Record High Efficiency Single-Walled Carbon Nanotube/Silicon p−n Junction Solar Cells. Nano Lett. 13, 96–99 (2013). 10.1021/nl3035652 23237412

[b12] Li X. . Role of HF in Oxygen Removal from Carbon Nanotubes: Implications for High Performance Carbon Electronics. Nano Lett. 14, 6179−6184 (2014). 25286024 10.1021/nl502401c

[b13] Pintossi C. . Direct Evidence of Chemically Inhomogeneous, Nanostructured, Si–O Buried Interfaces and Their Effect on the Efficiency of Carbon Nanotube/Si Photovoltaic Heterojunctions. J. Phys. Chem. C 117, 18688–18696 (2013).

[b14] Jia Y. . Achieving High Efficiency Silicon-Carbon Nanotube Heterojunctions Solar Cells by Acid Doping. Nano Lett. 11, 1901–1095 (2011). 21452837 10.1021/nl2002632

[b15] Wang F. . Enhancement Mechanism of the Photovoltaic Conversion Efficiency of Single-Walled Carbon Nanotube/Si Solar Cells by HNO_3_ Doping. Appl. Phys. Express 6, 102301–102304 (2013).

[b16] Del Gobbo . Silicon Spectral Response Extension Through Single Wall Carbon Nanotubes in Hybrid Solar Cells. J. Mater. Chem. C 1, 6752–6758 (2013).

[b17] Di J., Yong Z., Zheng X., Sun B. & Li B. Aligned Carbon Nanotubes for High Efficiency Schottky Solar Cells. Small 9, 1367–1372 (2013). 23463708 10.1002/smll.201202995

[b18] Li X. . Improved Efficiency of Smooth and Aligned Single Walled Carbon Nanotube/Silicon Hybrid Solar Cells. Energy Environ. Sci. 6, 879–887 (2013).

[b19] Ong P. L., Euler W. B. & Levitsky I. A. Hybrid Solar Cells Based on Single-Walled Carbon Nanotubes/Si Heterojunctions. Nanotechnology 21, 105203 (2010). 20157233 10.1088/0957-4484/21/10/105203

[b20] Collins P. G., Bradley K., Ishigami M. & Zettl A. Extreme Oxygen Sensitivity of Electronic Properties of Carbon Nanotubes. Science 287, 1801−1804 (2000). 10710305 10.1126/science.287.5459.1801

[b21] Goldoni A. . Spectroscopic characterization of contaminants and interaction with gases in single-walled carbon nanotubes. Carbon 42, 2099−2112 (2004).

[b22] Chopra S., McGuire K., Gothard N., Rao A. M. & Pham A. Selective gas detection using a carbon nanotube sensor. Appl Phys Lett 83, 2280 (2003).

[b23] Someya T., Small J., Kim P., Nuckolls C. & Yardley J. T. Alcohol vapour sensors based on single-walled carbon nanotube field effect transistors. Nano Lett 3, 877−881 (2003).

[b24] Anoshkin I. V. . Single-walled carbon nanotube networks for ethanol vapor sensing applications. Nano Research 6, 77−86 (2013).

[b25] Novak J. P. . Nerve agent detection using networks of single-walled carbon nanotubes. Appl Phys Lett 83, 4026 (2003).

[b26] Picaud F., Girardet C. & Rao A. M. A comparative study of single- and multiwalled carbon nanotube sensitivity to ammonia. J Appl Phys 105, 014315 (2009).

[b27] Vichchulada P., Zhang P. Q. & Lay M. D. Recent progress in chemical detection with single-walled carbon nanotube networks. Analyst 132, 719−723 (2007). 17646869 10.1039/b618824a

[b28] Kauffman D. R. & Star A. Carbon nanotube gas and vapour sensors. Angew Chem 47, 6550−6570 (2008). 18642264 10.1002/anie.200704488

[b29] Penza M., Rossi R., Alvisi M., Signore M. A. & Serra E. Effects of reducing interferers in a binary gas mixture on NO_2_ gas adsorption using carbon nanotube networked films based chemiresistors. J Phys D Appl Phys 42, 072002 (2009).

[b30] Zhang T., Mubeen S., Myung N. V. & Deshusses M. A Recent progress in carbon nanotube-based gas sensors. Nanotechnology 19, 332001 (2008). 21730614 10.1088/0957-4484/19/33/332001

[b31] Goldoni A., Petaccia L., Lizzit S. & Larciprete R. Sensing gases with carbon nanotubes: a review of the actual situation. J Phys Condens Matter 22, 013001 (2010). 21386215 10.1088/0953-8984/22/1/013001

[b32] Penza M., Rossi R., Alvisi M. & Serra E., Metal-modified and vertically aligned carbon nanotube sensors array for landfill gas monitoring applications. Nanotechnology 21, 105501 (2010). 20154374 10.1088/0957-4484/21/10/105501

[b33] Chiesa M. . Development of low-cost ammonia gas sensors and data analysis algorithms to implement a monitoring grid of urban environmental pollutants. J Environ Monit 14, 1565−1575 (2012). 22517026 10.1039/c2em30102d

[b34] Chen G., Paronyan T. M., Pigas E. M. & Harutyunyan A. R. Enhanced gas sensing in pristine carbon nanotubes under continuous ultraviolet light illumination. Scientific Reports 2, 343 (2012). 22461974 10.1038/srep00343 PMC3315270

[b35] Cao Q. . Medium-scale carbon nanotube thin-film integrated circuits on flexible plastic substrates. Nature Letters 454, 495−502 (2008). 10.1038/nature07110 18650920

[b36] Sun D. . Flexible high-performance carbon nanotube integrated circuits. Nature Nanotechnology 6, 156−161 (2011). 10.1038/nnano.2011.1 21297625

[b37] Rigoni F. . Enhancing the sensitivity of chemiresistor gas sensors based on pristine carbon nanotubes to detect low-ppb ammonia concentrations in the environment. Analyst 138, 7392−7399 (2013). 24171188 10.1039/c3an01209c

[b38] Zhao J., Buldum A., Han J. & Lu J. P. Gas molecule adsorption in carbon nanotubes and nanotube bundles. Nanotechnology 13, 195−200 (2002).

[b39] Bradley K., Gabriel J. C. P., Briman M., Star A. & Gruner G. Charge Transfer from Ammonia Physisorbed on Nanotubes. Phys. Rev. Lett. 91, 218301 (2003). 14683342 10.1103/PhysRevLett.91.218301

[b40] Pintossi C. & Sangaletti L. Semiconducting Carbon Nanotubes: Properties, Characterization and Selected Applications in Low-Dimensional and Nanostructured Materials and Devices. 239–259, Springer (2016).

[b41] Kong J. . Nanotube molecular wires as chemical sensors. Science 287, 622–625 (2000). 10649989 10.1126/science.287.5453.622

[b42] Chang Y. W., Oh J. S., Yoo S. H., Choi H. H. & Yoo K.-H. Electrically refreshable carbon-nanotube-based gas sensors. Nanotechnology, 18, 435504–7 (2007).

[b43] Uchida K. & Okada S. Electronic properties of a carbon nanotube in a field-effect transistor structure: A first-principles study. Phys. Rev B 79, 085402 (2009).

[b44] Pintossi C. . Steering the Efficiency of Carbon Nanotube–Silicon Photovoltaic Cells by Acid Vapor Exposure: A Real-Time Spectroscopic Tracking, ACS applied materials & interfaces 7, 9436–9444 (2015). 25902284 10.1021/am508973b

[b45] De Nicola F. . Controlling the thickness of carbon nanotube random network films by the estimation of the absorption coefficient. Carbon 95, 28–33 (2015).

[b46] Bai X. . The influence of gas absorption on the efficiency of carbon nanotube/Si solar cells, Appl. Phys. Lett. 102, 143105 (2013).

[b47] Fan G. . Hybrid effect of gas flow and light excitation in carbon/silicon Schottky solar cells. J. Mater. Chem. 22, 3330–3334 (2012).

[b48] Wang Z. L., Chen J. & Lin L. Progress in triboelectric nanogenerators as a new energy technology and self-powered sensors. Energy Environ. Sci. 8, 2250–2282 (2015).

[b49] Wang Z. L. & Wu W. Z. Nanotechnology-enabled energy harvesting for self powered micro-/nanosystems, Angew. Chem. Int. Edit. 51, 11700–11721 (2012). 10.1002/anie.201201656 23124936

[b50] Somani P. R. Pressure sensitive multifunctional solar cells using carbon nanotubes, Applied Physics Letters 96, 173504 (2010).

[b51] Sun H. . Flexible carbon nanotube/mono-crystalline Si thin-film solar cells, Nanoscale Research Letters 9, 514 (2014). 25258617 10.1186/1556-276X-9-514 PMC4174534

[b52] De Nicola F. . Record efficiency of air-stable multi-walled carbon nanotube/silicon solar cells. Carbon 101, 226–223 (2016).

[b53] Nelson J. The physics of Solar Cells. Imperial College Press (2003).

[b54] Luque A. & Hughes S. “*Handbook of Photovoltaic Science and Engineering*”. (John Wiley & Sons, 2006).

[b55] Sze S. Physics of Semiconductor Devices 2^nd^ Edition (John Wiley & Sons, 1981).

[b56] Rigoni F. . Gas sensing at the nanoscale: engineering SWCNT-ITO nano-heterojunctions for the selective detection of NH_3_ and NO_2_ target molecules. Nanotechnology 28, 035502 (2017). 27966471 10.1088/1361-6528/28/3/035502

[b57] Le Borgne V. . Enhanced UV photoresponse of KrF-laser-synthesized single-wall carbon nanotubes/n-silicon hybrid photovoltaic devices. Nanotechnology 23 215206 (2012). 22551529 10.1088/0957-4484/23/21/215206

[b58] Del Gobbo S. . Carbon nanotube semitransparent electrodes for amorphous silicon based photovoltaic devices. Appl. Phys. Lett. 98, 183113 (2011).

[b59] De Nicola F. . 100% internal quantum efficiency in polychiral single-walled carbon nanotube bulk heterojunction/silicon solar cells. Carbon 114, 402–410 (2017).

[b60] Han J. W., Kim B., Kobayashi N. P., Li J. & Meyyappan M. A simple method for the determination of doping type in nanomaterials based on electrical response to humidity. Appl Phys Lett. 101, 142110 (2012).

[b61] Ponpon J. P. & Siffert P. Open-circuit voltage of MIS silicon solar cells. Journal of Applied Physics 47, 3248–3251 (1976).

[b62] Chattopadhyay P. Functional dependence of open circuit voltage on interface parameters and doping concentration of MIS solar cells. phys. Stat. sol. (a) 140, 587–592 (1993).

[b63] Tans S. J., Verschueren A. R. M. & Dekker C. Room-temperature transistor based on a single carbon nanotube. Nature 393 49–52 (1998).

[b64] Wei J. . Double-Walled Carbon Nanotube Solar Cells. Nano Lett. 7, 2317–2321 (2007). 17608444 10.1021/nl070961c

