# Supplementary material for: A cross-functional nanostructured platform based on carbon nanotube-Si hybrid junctions: where photon harvesting meets gas sensing
Source: Sci Rep. 2017 Mar 15;7:44413. doi: 10.1038/srep44413 (PMC5353639; doi:10.1038/srep44413)
Supplement: Supplementary Information [file srep44413-s1.pdf]

Supplementary Information to:

A cross-functional nanostructured platform based on carbon nanotube-Si hybrid junctions: where photon harvesting meets gas sensing

F. Rigoni [(1,2)], C. Pintossi [(1)], G. Drera [(1)], S. Pagliara [(1)], G. Lanti [(1)],
P. Castrucci [(3)], M. De Crescenzi [(3)], L. Sangaletti [(1)]

[(1)] Surface Science and Spectroscopy Lab @ I-Lamp and Dipartimento di Matematica e Fisica, Università Cattolica del Sacro Cuore, Brescia, Italy
[(2)] CNR-INO and Sensor Lab, Dept. of Information Engineering, University of Brescia, Italy
[(3)] Dipartimento di Fisica, Università di Roma, Tor Vergata, Italy

Figure S1 shows the V_{OC} variation (ΔV_{OC}) during a sequence of exposures of Cell 55 to NH_3, acetone, and ethanol. To check the response characteristics at different scales, exposures to NH_3 have been carried out with different concentrations and time lengths. Seven different exposures are shown. Initially (1) Cell 55 was exposed to 10 ppm NH_3 and than (2) to 45 ppm NH_3. During the recovery from this exposure, Cell 55 was further exposed (3) to 5 ppm NH_3. Once full recovery was reached, the cell was exposed (4) to 20 ppm NH_3. Following this treatment, the cell was exposed to 30 ppm ethanol (5) and 30 pm acetone (6). For these exposures the response was negligible as compared to those obtained from NH_3. This shows that the cell response has a selectivity towards these molecules. Finally the cell was exposed (7) to 30 ppm NH_3. In the case of NH_3, the V_{OC} always showed a reduction proportional to the NH_3 concentration and recovery at room temperature was always attained.

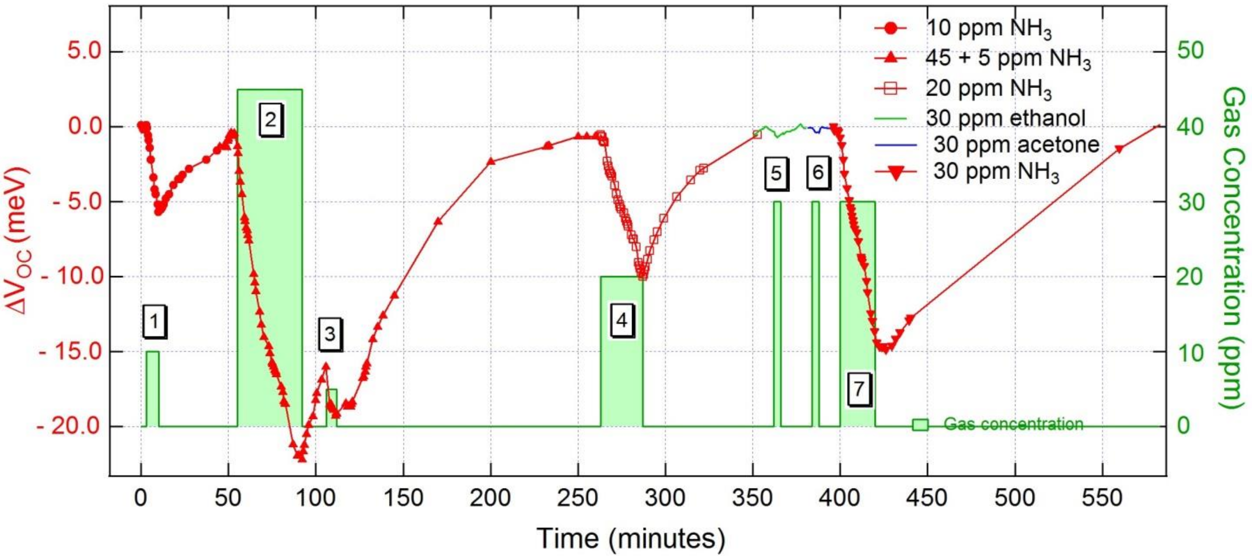

Figure S1. V_{OC} variation ((ΔV_{OC}, mV) upon Cell 55 exposure to different concentrations (ppm) of NH_3, ethanol and acetone.

Figure S2 shows the effects on the I-V and P-V curves of the change of R_S and R_{Sh} loads applied to Cell 15 (Fig.S2-a, left column) and Cell 55 (Fig.S2-b, right column). In both cases, the I_{sc} value is found to decrease as R_s increases from 0 Ohm to about 1 KOhm. On the other hand, V_{OC} is found to increase with the increase of R_{Sh}.

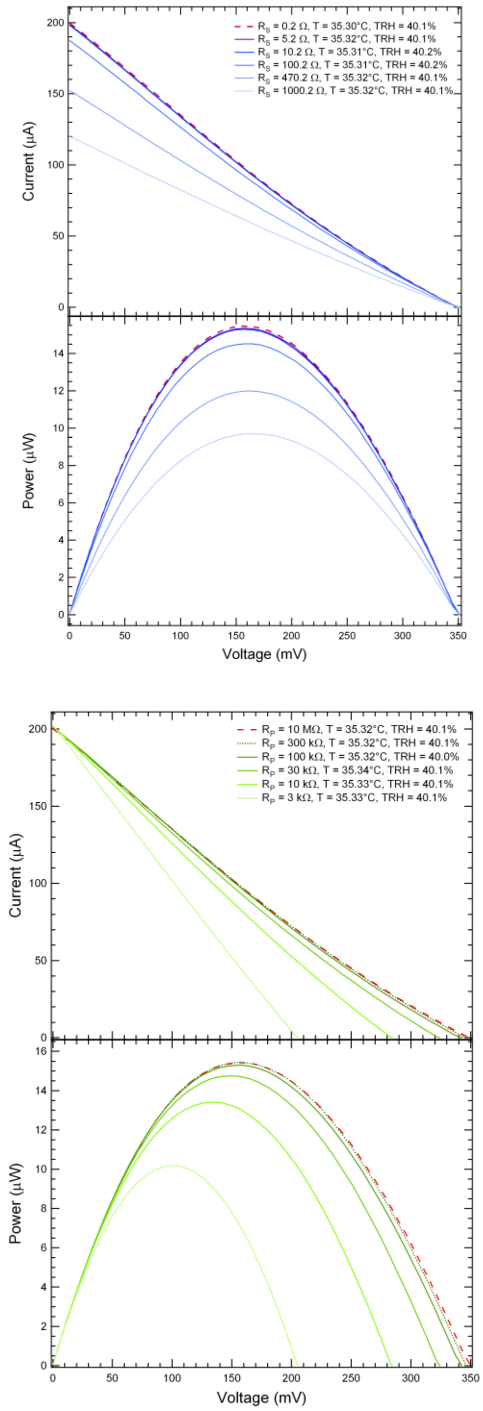

Fig.S2-a. Cell 15. Top panel: I-V and IV-V curves at different R_S values. Botton panel: I-V and IV-V curves at different R_{Sh} values.

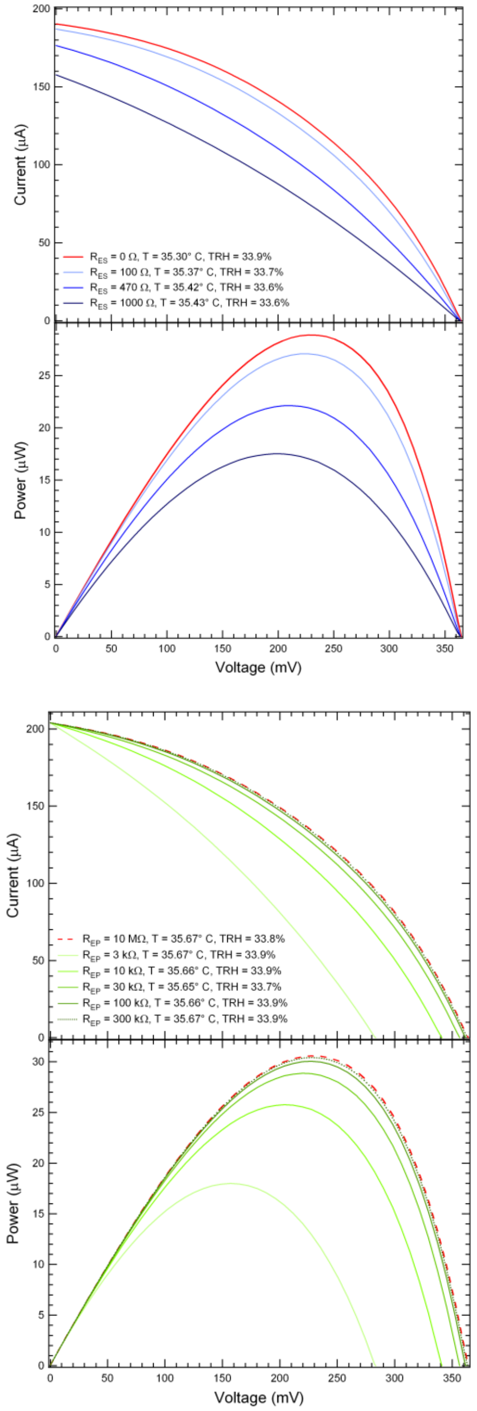

Fig. S2-b. Cell 55. Top panel: I-V and IV-V curves at different R_S values. Botton panel: I-V and IV-V curves at different R_{Sh} values.
